# Intramuscular coherence enables robust assessment of modulated supra-spinal input in human gait: an inter-dependence study of visual task and walking speed

**DOI:** 10.1007/s00221-023-06635-4

**Published:** 2023-05-18

**Authors:** Freschta Zipser-Mohammadzada, Marjelle Fredie Scheffers, Bernard A. Conway, David M. Halliday, Carl Moritz Zipser, Armin Curt, Martin Schubert

**Affiliations:** 1 Department of Neurophysiology, Spinal Cord Injury Center, Balgrist University Hospital, Zurich, Switzerland; 2 Faculty of Medicine, Utrecht University, Utrecht, The Netherlands; 3 Biomedical Engineering, University of Strathclyde, Glasgow, G4 0NW UK; 4 School of Physics, Engineering and Technology, University of York, York, YO10 5DD UK; 5 York Biomedical Research Institute, University of York, York, UK

**Keywords:** Visually guided walking, Intramuscular coherence, Gait, Reliability, Agreement, Test–retest, Motor control

## Abstract

Intramuscular high-frequency coherence is increased during visually guided treadmill walking as a consequence of increased supra-spinal input. The influence of walking speed on intramuscular coherence and its inter-trial reproducibility need to be established before adoption as a functional gait assessment tool in clinical settings. Here, fifteen healthy controls performed a normal and a target walking task on a treadmill at various speeds (0.3 m/s, 0.5 m/s, 0.9 m/s, and preferred) during two sessions. Intramuscular coherence was calculated between two surface EMG recordings sites of the Tibialis anterior muscle during the swing phase of walking. The results were averaged across low-frequency (5–14 Hz) and high-frequency (15–55 Hz) bands. The effect of speed, task, and time on mean coherence was assessed using three-way repeated measures ANOVA. Reliability and agreement were calculated with the intra-class correlation coefficient and Bland–Altman method, respectively. Intramuscular coherence during target walking was significantly higher than during normal walking across all walking speeds in the high-frequency band as obtained by the three-way repeated measures ANOVA. Interaction effects between task and speed were found for the low- and high-frequency bands, suggesting that task-dependent differences increase at higher walking speeds. Reliability of intramuscular coherence was moderate to excellent for most normal and target walking tasks in all frequency bands. This study confirms previous reports of increased intramuscular coherence during target walking, while providing first evidence for reproducibility and robustness of this measure as a requirement to investigate supra-spinal input.

*Trial registration Registry number/ClinicalTrials.gov Identifier*: NCT03343132, date of registration 2017/11/17.

**Supplementary Information:**

The online version contains supplementary material available at 10.1007/s00221-023-06635-4.

## Introduction

Human gait is highly adaptable allowing over-ground bipedal progression to remain effective even under challenging underfoot conditions. Continuous dynamic integration of multimodal sensory inputs and supra-spinal commands are required for anticipatory gait modifications during challenging walking (Friston 2000; Matthis et al. 2017; Halliday et al. 2003). Furthermore, precise foot placement and adaptive stepping are crucial for balance coordination and are mediated by supra-spinal control (Nielsen 2003; Yokoyama et al. 2019) and proprioceptive feedback mechanisms. In individuals with incomplete spinal cord injury (iSCI), damage affecting spinal tracts impairs the normal descending and ascending neural drive necessary for sensorimotor integration and consequently leads to a reduced capacity for gait adaptation including its visuomotor coordination (Barthélemy et al. 2010; Zipser-Mohammadzada et al. 2022). The quantification of walking impairment in patients with iSCI is difficult, given the ceiling effects evident in established clinical scores and tests (Bolliger et al. 2018). Quantification of walking impairment is necessary to assess walking performance and capacity to track rehabilitation and interventional effects (Pearson et al. 2004).

For this purpose, novel methods are required.

Aspects of sensorimotor integration during challenging walking can be assessed non-invasively through the use of coherence analysis by measuring either EMG coupling or the coupling between regional EEG and EMG signals (Boonstra et al. 2009b). Coherence measures quantify the strength of the coupling between two simultaneously recorded signals in the frequency domain (Halliday et al. 1995). Cortico-muscular coherence reflects the coupling between cortical and muscle activity, intra- and intermuscular coherence quantify the coupling within the same muscle and between separated muscles, respectively. The modulation of coherence measures obtained in physiologically relevant frequency bands provides information on the structure of oscillatory common neural drive to coactive muscles.

Intramuscular coherence in the high-frequency bands, such as beta-(15–32 Hz) and gamma-band (35–60 Hz), strongly associates with oscillatory neural activity related to supra-spinal drive to the spinal cord during voluntary movements. This was previously demonstrated in cortico-muscular coherence studies in the upper (Conway et al. 1995; Halliday et al. 1998; Salenius et al. 1997; Baker 2007; Kristeva et al. 2007; Kilner et al. 2000) and in lower limb muscles during walking (Petersen et al. 2012; Jensen et al. 2018; Roeder et al. 2018; Charalambous and Hadjipapas 2022) and ankle dorsiflexion tasks (Perez et al. 2006; Spedden et al. 2019b).

Intramuscular coherence in the alpha-band (8–12 Hz) may not only be modulated by EMG envelope and gait rhythm features (Halliday et al. 2003) but is also associated with spinal and subcortical systems as suggested by studies investigating coherence in lower (Norton et al. 2003; Aguiar et al. 2018) and upper limb muscles (Boonstra et al. 2009a, 2009b; Grosse and Brown 2003). The alpha-band coherence increases in response to bilateral muscle activity (Boonstra et al. 2009a, 2009b) or startle reflexes representing the subcortical system (Grosse and Brown 2003), more specifically, the reticulo-spinal system. Regarding the association to the spinal systems, the alpha-band coherence, in contrast to the beta- and gamma-band, is usually unaffected in individuals with central neurological conditions, such as stroke or iSCI, in line with the notion that it is mainly deficits in the supra-spinal drive to the motoneuron pools that limit gait performance post-stroke or iSCI (Hansen et al. 2005; Zipser-Mohammadzada et al. 2022). Importantly, when studying visuomotor performance in tasks requiring enhanced attention and/or motor precision, increased high-frequency cortico-muscular coherence is reported (Perez et al. 2006; Kristeva et al. 2007; Kristeva-Feige et al. 2002; Feige et al. 2000) suggesting that coherence in this frequency band may serve as a marker for changes in supra-spinal control during challenging motor tasks. Although, it needs to be highlighted that other neural circuits, such as ascending sensory tracts (Riddle and Baker 2005; Witham et al. 2011), might also influence high-frequency cortico-muscular coherence.

Several studies have demonstrated that intramuscular coherence was increased during a target walking task compared to normal walking (Jensen et al. 2018; Spedden et al. 2019a).

We have shown previously that, when compared to normal walking, a challenging visually guided walking task is associated with enhanced high-frequency coherence (21–44 Hz) in controls, but not in patients with severe iSCI (Zipser-Mohammadzada et al. 2022). We found that regulating the preferred walking speed, i.e., walking slower, was one mechanism to adapt and adjust to the visually guided target walking task (TW). However, this was variable across subjects, and a systematic relationship between intramuscular coherence and walking speed could not be determined (Zipser-Mohammadzada et al. 2022). Of note, walking speed is known to have strong effects on kinematics and EMG modulation during gait (Kibushi et al. 2018; Fukuchi et al. 2019) as well as a surmised association with changing attention which likely would influence coherence when assessed during a gait paradigm.

To investigate speed-related changes during gait further, this study aimed to examine the speed-dependent modulation of intramuscular coherence during TW compared to normal walking (NW) and the robustness of the TW effect (as a modulatory intervention) in a healthy subject cohort. Furthermore, to investigate the clinical utility of intramuscular coherence as a surrogate marker for supra-spinal input, the reproducibility over time was assessed with test–retest reliability and agreement measures. Both help assessing intersession variability. Reliability measures provide information on how well coherence modulation would be reproduced, and agreement presents how close the coherence estimates are for repeated measures taking measurement errors into account (Hernaez 2015). Therefore, both measures are important if coherence estimates are to be used in assessing patient’s recovery from neurological deficits or improving following rehabilitation intervention. Previously, the test–retest reliability of intramuscular coherence during overground and treadmill walking was shown to be moderate in young adults (Gennaro and de Bruin 2020; van Asseldonk et al. 2014). The present study provides first evidence that the intramuscular coherence increase seen during visually guided walking is robust, especially at preferred walking speeds and that intramuscular coherence during both tasks is reproducible. These findings have implications for our understanding of the relationship between coherence and the effects of walking speed in a young healthy cohort. They lend further support to the idea of coherence measures as a surrogate marker for the assessment functioning of supra-spinal control.

## Methods

### Participants

Control participants were recruited from December 2021 until March 2022 to take part in this prospective study. Informed consent was obtained from each participant prior to inclusion in the study. The participants were included if they were ≥ 18 years old. Exclusion criteria for participants included (1) history of neurological disease, (2) psychiatric or medical morbidity interfering with the experimental tasks, (3) orthopedic conditions, (4) heart insufficiency NYHA III–IV (New York Heart Association; heart insufficiency symptoms after light endurance, III, and permanent symptoms, IV), (5) COPD Gold III or IV (Global Initiative for Chronic Obstructive Lung Disease), and (6) dermatological concerns interfering with proper attachment of electrodes and sensors to the skin. The sample size was based on previous studies investigating test–retest reliability and agreement of coherence in control participants (Gennaro and de Bruin 2020; van Asseldonk et al. 2014). The current study was registered at ClincalTrials.gov (NCT03343132, date of registration 2017/11/17) and was approved by the Zurich cantonal ethics Committee (BASEC-NR. 2017-01780). All experiments were conducted in accordance with the Declaration of Helsinki.

### Experimental set-up

Participants took part in two experimental sessions, session 1 and session 2 (i.e., test and retest), which were separated by at least one week. The same researcher conducted both experimental sessions, and during each session, the same experimental procedure was applied.

### Experimental procedure

Within each session, participants walked on a synchronized dual-belt treadmill with two built-in force plates (GRAIL, Motek Medical B.V., The Netherlands) wearing their own athletic shoes. In the first experimental session, the participants’ preferred self-selected walking speeds were determined the same way for NW and TW using their respective task set-up (see below): the treadmill speed was increased by 0.1 m/s increments, while constantly receiving verbal feedback from the participant. When participants mentioned they were walking at their comfortable speed, the treadmill speed was increased again by 0.1 m/s. If this was too fast, the treadmill speed was decreased by 0.1 m/s. This way, the participants’ preferred walking speeds were confirmed. The preferred speeds were then used for both experimental sessions, sessions 1 and 2 (i.e., test and retest). The experiment in session 1 continued and in session 2 started with a five-minute warm-up during which participants walked on the treadmill to familiarize themselves with the treadmill, followed by a short (one minute) familiarization of the TW task (see below) with their previously determined preferred walking speed.

Next, the recordings for the experiment started, which comprised two tasks; the NW and the TW task. Each task lasted three minutes.

For the NW task, participants walked naturally while looking straight ahead at a green ball displayed on a screen in front of the treadmill.

For the TW task, participants stepped onto moving targets. The moving targets were presented in the form of white dots (diameter = 10 cm) displayed onto and in front of the treadmill belt (Mohammadzada et al. 2022). The speed of the moving targets, i.e., white dots, was equal to the treadmill speed, such that participants perceived them as stationary relative to the treadmill (Motek Forcelink, Amsterdam, the Netherlands, version 3.34.1). Participants were instructed to step with their second metatarsal bone (MT2) to the middle of the targets as precisely as possible while staying safe. Targets were distributed such that random adjustments of step length and width were required by the participant. The random distribution was identical for each participant. Variation of step length and width was based on a continuous uniform distribution randomization between 40 and 80% of a fixed 0.8 m step length and 0.25 m step width, respectively. The rationale was to cover step lengths–widths of people with different heights to create a similar situation to the outside world, where step lengths and widths also have to be adjusted, irrespective of one’s own step length and width.

Both the NW task and TW task were performed at four different treadmill speeds; three predetermined speeds of 0.3 m/s (NW03, TW03), 0.5 m/s (NW05, TW05), and 0.9 m/s (NW09, TW09) and the self-selected preferred speed (NWp, TWp). If the preferred speed was the same as a predetermined speed, that particular task was disregarded. In total, eight tasks had to be completed during each experimental session. During each experimental session, the eight tasks were performed in a random order to minimize carryover effects.

### Data acquisition

Demographics, including age, height, sex, and relevant medical history, were collected prior to the start of the experiment (see Table 1). During the experiments, two types of recordings were assessed. First, kinematic information was obtained through a passive infrared motion capture system (Vero, Vicon Motion Systems, LTd, Oxford, UK) at 100 Hz. Reflective markers were placed on the heel and second metatarsal bone (MT2) on the right and left leg to identify the heel strike (HS) and toe off (TO), respectively. Second, muscle activity was recorded through wireless surface EMG sensors (Myon AG, Switzerland) at 2000 Hz that were attached to disposable Ag/AgCl electrode pairs (Kendall H124SG) placed on the proximal (TAp) and distal (TAd) site of the Tibialis anterior muscle on both legs. The distance between the two EMG sensors on each leg was at least 10 cm to avoid cross-talk between the signals (Hansen et al. 2005).

**Table 1:** Participant demographics

ID	Age (years)	Sex (m/f)	Height (cm)	Walking speed (m/s)		Mean step length (m)				Mean step width (m)				Time between sessions (days)
						NWp		TWp		NWp		TWp		
				NWp	TWp	Session 1	Session 2	Session 1	Session 2	Session 1	Session 2	Session 1	Session 2	
1	43	m	172	0.8	0.8	0.51	0.51	0.4	0.4	0.09	0.12	0.15	0.17	7
2	42	m	183	1.2	1.2	0.71	0.69	0.4	0.4	0.1	0.14	0.24	0.23	7
3	24	f	158	0.95	0.9	0.57	0.56	0.4	0.4	0.07	0.09	0.15	0.17	7
4	30	m	188	1.3	0.9	0.73	0.74	0.39	0.41	0.11	0.11	0.17	0.19	7
5	34	f	170	1.1	0.9	0.65	0.66	0.41	0.41	0.1	0.09	0.21	0.2	7
6	35	f	160	1.05	0.9	0.63	0.62	0.4	0.4	0.08	0.08	0.19	0.2	7
7	27	m	192	1.1	1.05	0.72	0.72	0.4	0.4	0.12	0.1	0.2	0.21	7
8	32	f	158	1	0.9	0.56	0.57	0.4	0.4	0.12	0.11	0.21	0.21	7
9	27	f	165	1	1	0.63	0.62	0.4	0.4	0.07	0.06	0.16	0.17	7
10	29	m	183	1.35	0.95	0.75	0.74	0.4	0.4	0.08	0.11	0.16	0.15	7
11	27	m	183	1.3	1	0.8	0.79	0.4	0.4	0.08	0.1	0.2	0.2	7
12	35	f	171	1.4	0.9	0.75	0.75	0.39	0.4	0.13	0.15	0.24	0.24	7
13	29	f	170	1.3	1.05	0.73	0.75	0.39	0.4	0.14	0.14	0.24	0.24	12
14	26	f	171	1.2	1.1	0.72	0.7	0.41	0.39	0.07	0.07	0.19	0.19	7
15	29	m	190	1.3	1	0.73	0.72	0.4	0.4	0.09	0.08	0.2	0.21	7
Mean	31		173	1.16	0.97	0.68	0.68	0.4^a^	0.4^a^	0.1	0.1	0.19	0.2	7.33
SD	5.61		11.53	0.17	0.10	0.08	0.08	0^a^	0^a^	0.02	0.03	0.03	0.03	1.29
Stats				*p* < 0.05		*p* > 0.05		*p* > 0.05^b^		*p* > 0.05		*p* > 0.05		

Walking speed, mean step length, and step width are reported for each participant's preferred walking speed

ID, participant number; m, male; f, female; NWp, Normal walking preferred speed; TWp, Target walking preferred speed; SD, standard deviation; Stats, statistics for comparisons between variables, paired *t*-test

^a^Median and interquartile range

^b^*p*-value for the paired Wilcoxon test

### Data processing and analysis

Kinematic and EMG data were processed using Vicon Nexus 2.9.1 (Vicon, Oxford, UK) and Matlab R2019b (The MathWorks, Inc., Natick, MA, USA) software. From the kinematic data, the time of HS and TO was extracted by calculating the moment of zero velocity for the heel and toe marker, respectively (Zeni et al. 2008). Step length was calculated by taking the time in seconds between two consecutive heel contacts of the right and left foot, from the time of the right heel strike to the time of the left heel strike. This step time was then multiplied by walking speed which resulted in the step length in meters.

EMG data were bandpass-filtered at 10–300 Hz (zero-phase, second-order Butterworth) and full-wave rectified. Pre-processing of EMG with and without rectification is discussed in Farina et al. (2013), McClelland et al. (2012), Ward et al. (2013), and Halliday and Farmer (2010). We include the rectification pre-processing step to maximize the detection of motor unit firing patterns and suppress information related to the motor unit action potential (Farmer and Halliday 2014).

EMG recordings were checked for cross-talk, characterized by a narrow central peak in the cumulant density and significant high-amplitude broad-band coherence (Hansen et al. 2001; Halliday et al. 2003). No cross-talk between the EMG recordings was found.

Intramuscular coherence between the TAp and TAd of the right leg was analyzed for the swing phase using Type 1 spectral analysis (Halliday et al. 1995) (Neurospec 2.0, available at: https://neurospec.org/). In the Neurospec routines, discrete Fourier transformations (DFT) were applied to the data to decompose and analyze them in the frequency domain. The segment length for the DFT was based on the duration of the swing phase. The duration of each swing phase was defined based on the averaged, normalized EMG data of all participants throughout the gait cycle with respect to the HS and TO. Thus, the duration of the swing phase was defined as 100 ms before TO until 150 ms after HS. DFT segments used 4096 samples, which equals 2.048 s (2000 Hz sampling), giving a frequency resolution of 0.49 Hz, covering the entire swing phase of one gait cycle. Segments with greater than 4096 samples were truncated and those with fewer than 4096 samples were zero padded to give a common set of Fourier frequencies for comparative analyses. Swing phase durations in samples varied from 1300 to 3140 for NW tasks and from 800 to 4480 for TW tasks. For each 3-min recording of a task, the full recording was analyzed. The mean number of gait cycles analyzed for each task and two sessions was: NWp 152 (± 7.7); NW09 137 (± 9.4); NW05 98 (± 10.2); NW03 70 (± 10.9); TWp 216 (± 22.6); TW09 201 (± 2.1); TW05 111 (± 1.6); TW03 65 (± 1.4). These numbers equal the number of segments used for the coherence analysis.

Then, coherence analysis was performed between the two EMG signals (i.e., right TAp and TAd) during the swing phase for each task and each participant. Coherence quantifies the strength of a correlation between two signals in the frequency domain. For two EMG signals $x$ and $y$ at frequency $\lambda$, coherence is defined as the absolute square of the cross-spectrum between the two EMG signals $f_{xy}$, normalized by multiplying the auto-spectra of each EMG signal $\left( {f_{xx} ,f_{yy} } \right)$ (Halliday et al. 1995):

1 $$|R_{xy} (\lambda )|^{2} = \frac{{|f_{xy} \left( \lambda \right)|^{2} }}{{f_{xx} (\lambda )f_{yy} (\lambda )}}$$

This provides a value between 0 and 1 at frequency $\lambda$, where 0 indicates no linear relationship and 1 indicates a perfect linear relationship. Furthermore, coherence was significant when it was greater than the upper 95% confidence limit (CL), considering the number of the segments (L):

2 $$CL = 1 - (0.05)^{1/(L - 1)}$$

Pooled coherence provides a single population measure of coherence. It combines the data of individual participants and assumes that the data sets to be pooled are independent (Amjad et al. 1997). In this study, the pooled coherence was calculated for each task in each session, sessions 1 and 2 (i.e., test and retest) to visually obtain differences between tasks and between sessions at a population level. The $\chi^{2}$ extended difference of coherence test (Amjad et al. 1997) was used to explore differences at each frequency between tasks at both sessions and between sessions 1 and 2 within each task and the details are reported in the Online Resource 1.

### Statistical analysis

Statistical analysis was performed using RStudio version 2.1 (R Foundation for Statistical Computing, Austria), Matlab R2019b (The MathWorks, Inc., Natick, MA, USA) software, and SPSS (IBM SPSS Statistics for Macintosh, Version 28.0.1.1). Descriptive statistics were used to analyze the demographics of the study group. The Shapiro–Wilk test was used to test for normality, which was confirmed by inspection of quantile–quantile plots. To compare differences between groups, a two-sided paired Student’s *t *test was used for normally distributed data, and a two-sided paired Wilcoxon test for non-normally distributed data. Unless otherwise mentioned, data are reported as the mean and standard deviation (SD). A *p*-value less than 0.05 was considered significant. Using a data-driven approach, coherence values were averaged for the single participants for each task in the low-frequency band (5–14 Hz) and high-frequency band (15–55 Hz). These values were used for the ANOVA’s and reliability and agreement analysis: Two repeated measures ANOVA’s were performed separately for the mean low- and high-frequency band coherence with the within-subject factors speed (0.3 m/s, 0.5 m/s, 0.9 m/s, and preferred speed), condition (TW and NW), and time (sessions 1 and 2). For the ANOVA’s, a variance stabilizing transform of the coherence was used by applying Fisher’s transform tanh^−1^ of the magnitude coherency (Amjad et al. 1997). All reported *p*-values were Bonferroni-corrected.

Reliability of intramuscular coherence between the two sessions was described for each task by the intraclass correlation coefficient ($ICC (3,1)$), using a single-rating, absolute-agreement, 2-way mixed effects model. This calculation is based on the mean squares (i.e., estimates of population variances based on the variability among a given set of measures), and is performed using the following formula (Koo and Li 2016):

3 $$ICC \left( {3,1} \right) = \frac{{MS_{P} - MS_{E} }}{{MS_{P} + \left( {k - 1} \right) MS_{E } + \frac{k}{n}(MS_{S} - MS_{E} )}}$$

Here, $MS_{P}$ denotes the mean square for the participants, $MS_{E}$ the mean square for error, $MS_{S}$ the mean square for the sessions, $k$ the number of sessions, and $n$ the number of participants. Although there are no standard ICC values that define acceptable reliability, it is suggested that ICC values below 0.50 indicate poor reliability, values between 0.50 and 0.75 moderate reliability, values between 0.75 and 0.90 good reliability, and values greater than 0.90 excellent reliability (Koo and Li 2016).

Agreement of intramuscular coherence between the two sessions was described for each task using the Bland–Altman method (Bland and Altman 1986). This involves calculations of the bias (i.e., the mean difference of coherence between sessions) and the 95% lower and upper limits of agreement (LOA), defined as the bias ± 1.96 × SD. These LOA give an estimate of the interval in which 95% of the differences of intramuscular coherence in session 2 compared to session 1 are expected to fall.

## Results

### Participants and gait parameters

Fifteen healthy participants (seven males, eight females; age: 31 ± 5.6 years) were included in the current study (Table 1). The mean preferred speed for NW (NWp) was 1.16 m/s (± 0.17), which was significantly greater than the mean preferred speed for TW (TWp) of 0.97 m/s (± 0.10) (*p* < 0.001). The mean step length was not significantly different between session 1 and 2 for NWp (both 0.68 m ± 0.08, *p* = 0.29), along with the median step length for TWp (Mdn = 0.40 m, IQR = 0, *p* = 0.71). Similarly, the mean step width was not significantly different between sessions 1 and 2 for NWp (0.10 m ± 0.02 and 0.10 m ± 0.03, respectively, *p* = 0.18) and TWp (0.19 m ± 0.03 and 0.20 m ± 0.03, respectively, *p* = 0.11). However, predictably, the mean step length was significantly different between NWp and TWp during both session 1 (*p* < 0.001) and session 2 (*p* < 0.001). The same holds for the step width, which was significantly different between NWp and TWp in session 1 (*p* < 0.001) and session 2 (*p* < 0.001). Time between the two sessions was seven days for all participants except for one, due to changes in availability of the participant.

### Intramuscular coherence during normal and target walking

**Fig. 1:**
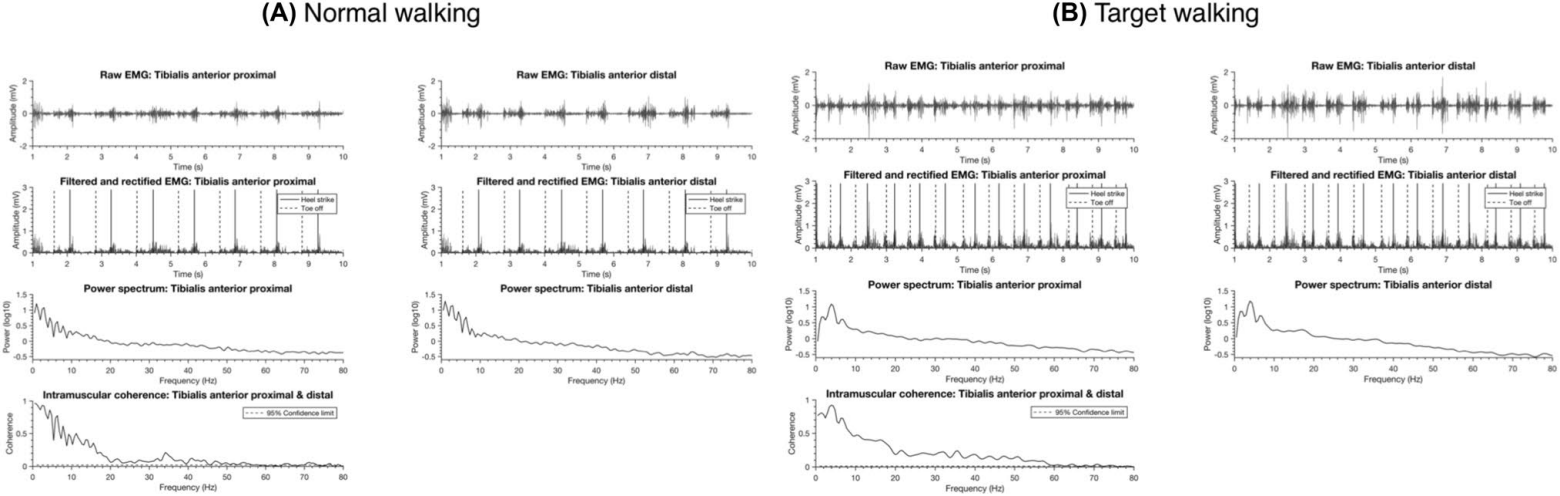
Raw and processed EMG signals during Normal walking (**A**) and Target walking (**B**) for a single participant (ID 14) at their preferred speed. In the first row, 10-s samples of raw EMG data from the Tibialis anterior proximal and distal are depicted. In the second row, the respective band-pass-filtered and full-wave-rectified EMG data are shown with heel strike and toe off events. The corresponding power spectra of the full 3-min EMG recordings are displayed in the third row. The intramuscular coherence is derived from these EMG recordings shown in the last row

**Fig. 2:**
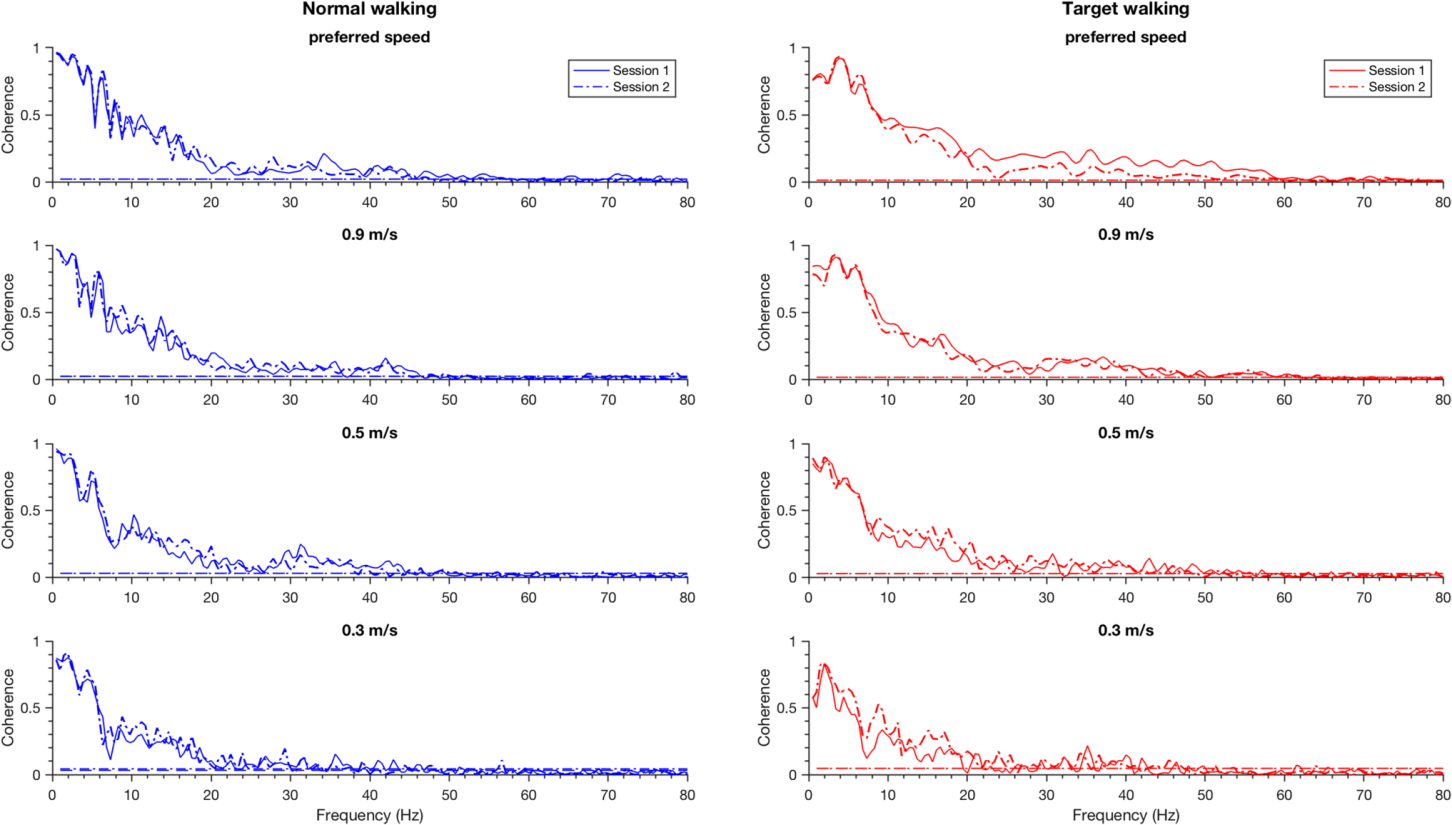
Intramuscular coherence between the Tibialis anterior proximal and distal during various tasks for a single participant (ID 14). Intramuscular coherences during Normal walking (left, blue) and Target walking (right, red) during different speeds (from top to bottom: preferred speed, 0.9 m/s, 0.5 m/s, and 0.3 m/s) for Session 1 (solid) and Session 2 (dashed) are shown

A representative example of raw EMG recordings of a single participant (ID 14) is shown in Fig. 1, along with the processed EMG data and the spectral and coherence analysis for NW (Fig. 1a) and TW (Fig. 1b). This figure illustrates both power and coherence for this single participant and highlights the role of coherence as a normalized measure, Eq. 1, in assessing common supra-spinal drive to motoneurons. For this participant, intramuscular coherence between TAp and TAd for each task and session is presented in Fig. 2. All participants showed significant intramuscular coherence in the low-frequency (5–14 Hz) and high-frequency band (15–55 Hz) in all tasks, during each session.

**Fig. 3:**
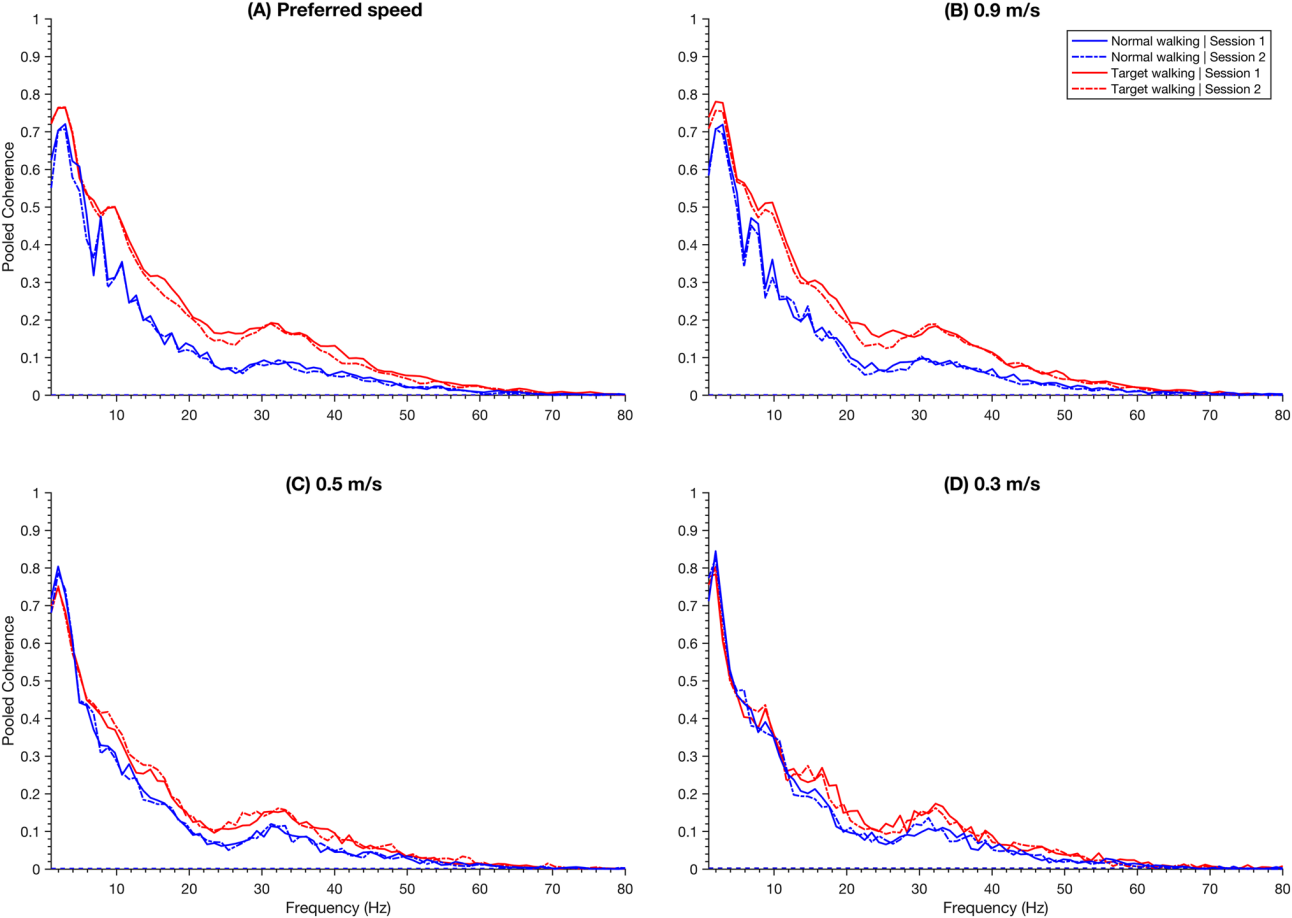
Pooled coherence between the Tibialis anterior proximal and distal shown for different speeds. The pooled coherences are shown for Normal walking (blue) and Target walking (red) in Session 1 (solid) and Session 2 (dashed) during preferred walking speed (**A**), 0.9 m/s (**B**), 0.5 m/s (**C**) and 0.3 m/s (**D**). The pooled coherence is a representative measure of all participants (n = 15). Dashed lines represent the 95% significance limit of each task (they appear as one line since they are very similar)

**Fig. 4:**
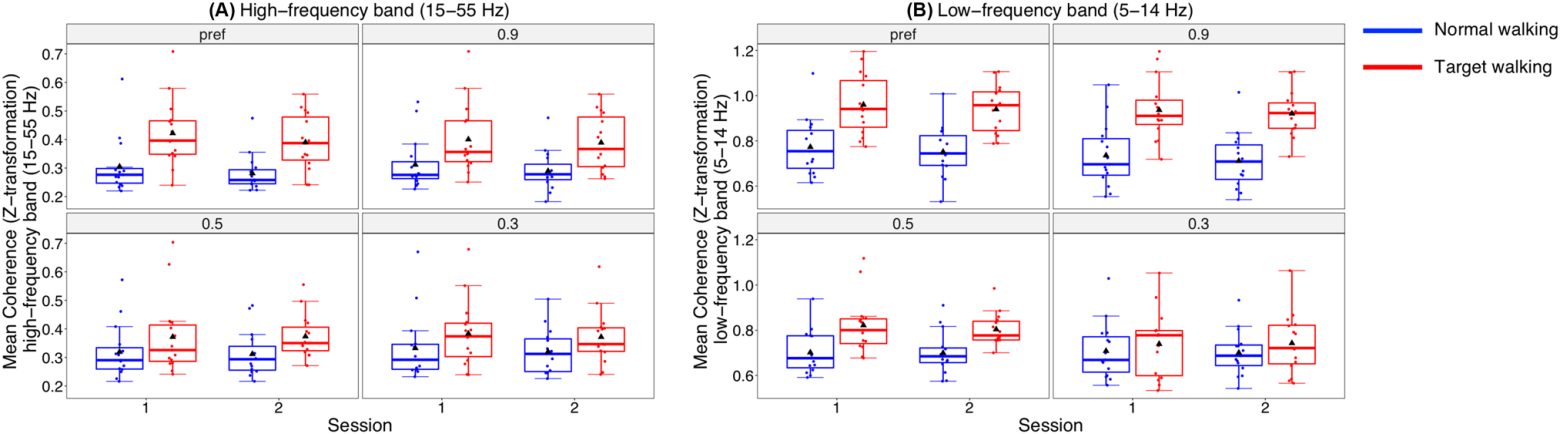
Speed- and task-specific modulation of mean intramuscular coherence. Intramuscular Tibialis anterior proximal and distal coherence is shown for Session 1 and 2 for the high-frequency (**A**) and low-frequency (**B**) bands for Normal walking (blue) and Target walking (red). An interaction effect is obtained between speed (preferred speed, 0.9 m/s, 0.5 m/s, and 0.3 m/s) and task (Target walking, Normal walking). Higher coherence is obtained for Target walking compared to Normal walking for almost all speeds and frequency bands. With increased walking speed, the difference between Target and Normal walking becomes greater. For more details see Results. pref = preferred

Additionally, pooled coherence is shown for each task and session in Fig. 3 to visualize the modulation across the frequencies and speeds. Details on significant differences in the pooled coherences between tasks and sessions are reported in the Online Resource 1. The modulations are quantified by calculating separate ANOVA’s for each frequency band. Three-way repeated measures ANOVA’s were performed to separately analyze the effect of speed, task, and time on intramuscular low- and high-frequency coherence (Fig. 3). The repeated measures ANOVA for the high-frequency coherence revealed a statistically significant interaction between speed and task (F(3, 42) = 12.13, *p* < 0.001) (Fig. 4a). Simple main effects analysis showed that intramuscular high-frequency coherence during TW was significantly higher than during NW at all speed levels, between TW03 and NW03 (*p* < 0.001), TW05 and NW05 (*p* < 0.001), TW09 and NW09 (*p* < 0.001), and TWp and NWp (*p* < 0.001). Furthermore, intramuscular high-frequency coherence during TW showed that TWp was significantly higher than TW05 (*p* = 0.032).

Intramuscular high-frequency coherence during NW was not affected by speed (*p* > 0.05).

The repeated measures ANOVA for the low-frequency coherence with a Greenhouse–Geisser correction showed a statistically significant interaction between the effects of speed and task (F(1.59, 22.19) = 11.52, *p* < 0.001) (Fig. 4b). Simple main effects analysis showed that the intramuscular low-frequency coherence during TW was significantly higher than during NW at almost all speed levels, between TW05 and NW05 (*p* < 0.001), TW09 and NW09 (*p* < 0.001), and TWp and NWp (*p* = 0.003), but not between TW03 and NW03 (*p* = 0.192). Furthermore, intramuscular low-frequency coherence during TW depended on speed, revealing that the intramuscular coherence during TW09 was higher than during TW03 (*p* < 0.001) and TW05 (*p* < 0.001), and intramuscular coherence during TWp was higher than during TW03 (*p* < 0.001) and TW05 (*p* < 0.001). Intramuscular coherence during TW05 was higher than TW03 (*p* = 0.014) and no difference was found between TW09 and TWp (*p* > 0.05) intramuscular low-frequency coherence. In NW, only intramuscular low-frequency coherence during NWp was significantly higher than NW09 (*p* = 0.024).

### Reliability of intramuscular coherence between sessions

Reliability of high- and low-frequency coherence is presented by ICC values in Table 2A and B, respectively. The high-frequency coherence showed “moderate to excellent” reliability for NW03, TW05, NW09, TW09, NWp, and TWp. TW03 and NW05 had “good to excellent” reliability.

**Table 2:** Reliability of intramuscular coherence

Task	ICC	95% confidence interval		*F* test with true value 0		
		Lower bound	Upper bound	Value	*df*	Sig
(A) High-frequency band (15–55 Hz) coherence						
NW03	0.82	0.55	0.94	10.16	14	< 0.0001
TW03	0.95	0.86	0.98	39.41	14	< 0.0001
NW05	0.92	0.77	0.97	23.10	14	< 0.0001
TW05	0.81	0.53	0.93	9.56	14	< 0.0001
NW09	0.88	0.67	0.96	15.29	14	< 0.0001
TW09	0.87	0.66	0.95	14.36	14	< 0.0001
NWp	0.87	0.66	0.96	14.61	14	< 0.0001
TWp	0.85	0.62	0.95	12.69	14	< 0.0001
(B) Low-frequency band (5–14 Hz) coherence						
NW03	0.80	0.50	0.93	8.90	14	< 0.0001
TW03	0.92	0.77	0.97	23.42	14	< 0.0001
NW05	0.84	0.60	0.94	11.77	14	< 0.0001
TW05	0.75	0.40	0.91	6.99	14	< 0.0001
NW09	0.84	0.58	0.92	11.28	14	< 0.0001
TW09	0.89	0.71	0.96	17.79	14	< 0.0001
NWp	0.79	0.48	0.92	8.38	14	< 0.0001
TWp	0.91	0.75	0.97	21.15	14	< 0.0001

Reliability of high-frequency (15–55 Hz) (A) and low-frequency (5–14 Hz) (B) coherence during various tasks, comparing Session 1 and 2. Reliability is represented by the intraclass correlation coefficient (ICC) using a single-rating, absolute-agreement, 2-way mixed effects model

NW03, Normal walking 0.3 m/s; TW03, Target walking 0.3 m/s; NW05, Normal walking 0.5 m/s; TW05, Target walking 0.5 m/s; NW09, Normal walking 0.9 m/s; TW09, Target walking 0.9 m/s; NWp, Normal walking preferred speed; TWp, Target walking preferred speed; df, degrees of freedom; Sig, significance

Reliability of the low-frequency coherence showed slightly lower ICC values overall, reliability for NW03, TW05, and NWp was “poor to excellent”. For the tasks, NW05, NW09, and TW09 it was “moderate to excellent”, and for TW03 and TWp it was “good to excellent”.

### Agreement of intramuscular coherence

**Fig. 5:**
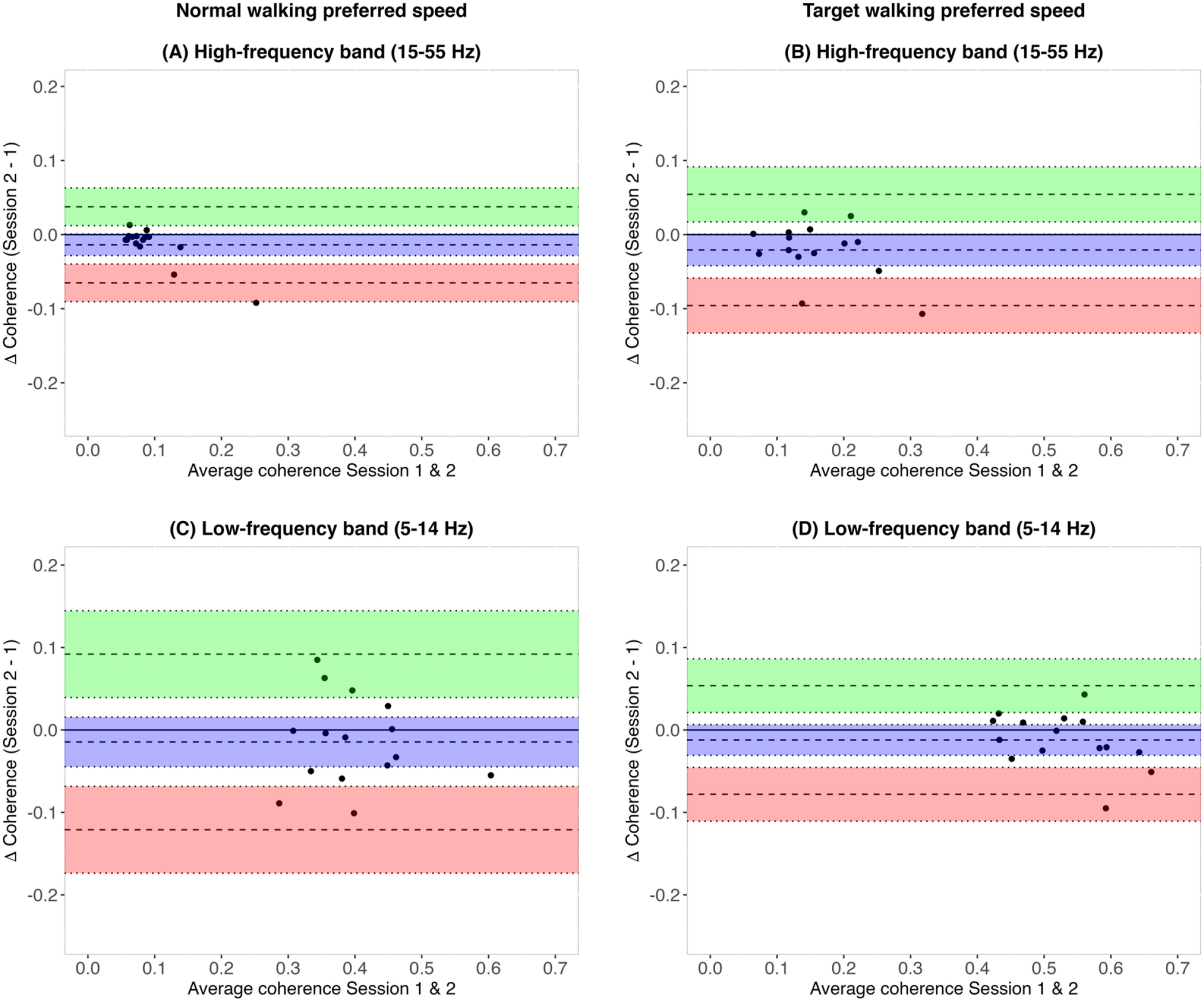
Bland–Altman plots for comparison of intramuscular coherence between Session 1 and Session 2. Bland–Altman plots show the agreement of intramuscular coherence of the Tibialis anterior proximal and distal between Session 1 and Session 2 at participants’ preferred speed. Each dot represents a single participant (n = 15). The left column depicts the agreement for the Normal walking task for high-frequency (15–55 Hz) (**A**) and low-frequency (5–14 Hz) (**C**) coherence, whereas the right column shows the agreement for the Target walking task for high-frequency (15–55 Hz) (**B**) and low-frequency (5–14 Hz) (**D**) coherence. The bias and its confidence limits are shown (blue), along with the upper (green) and lower (red) limits of agreement and its confidence limits

Agreement of intramuscular high- and low-frequency coherence was presented with the bias and limits of agreement (LOA), shown in Table 3A and B, respectively. No significant systematic differences were found for any of the tasks (Table 3A and B). Further visualization of agreement at the preferred speed for NW and TW is presented with Bland–Altman plots (Fig. 5). For all frequency bands, except for the NW high-frequency band (Fig. 5a), measurements of all participants fall within the LOA, meaning the data is not erratically variable.

**Table 3:** Agreement of intramuscular (A) high-frequency (15–55 Hz) and (B) low-frequency (5–14 Hz) coherence between Session 1 and 2

Task	Bias		Limits of agreement	
	Value	95% CI	Lower	Upper
(A) High-frequency band (15–55 Hz) coherence				
NW03	− 0.008	[− 0.029 0.013]	− 0.083	0.066
TW03	− 0.011	[− 0.023 0.001]	− 0.053	0.032
NW05	− 0.006	[− 0.018 0.006]	− 0.048	0.036
TW05	− 0.003	[− 0.028 0.022]	− 0.092	0.087
NW09	− 0.013	[− 0.026 0.001]	− 0.060	0.034
TW09	− 0.009	[− 0.030 0.012]	− 0.083	0.065
NWp	− 0.014	[− 0.028 0.001]	− 0.065	0.038
TWp	− 0.021	[− 0.042 0.0004]	− 0.096	0.054
(B) Low-frequency band (5–14 Hz) coherence				
NW03	− 0.005	[− 0.035 0.025]	− 0.110	0.10
TW03	0.003	[− 0.021 0.026]	− 0.080	0.085
NW05	− 0.002	[− 0.024 0.020]	− 0.080	0.080
TW05	− 0.011	[− 0.038 0.017]	− 0.110	0.088
NW09	− 0.018	[− 0.047 0.011]	− 0.121	0.086
TW09	− 0.009	[− 0.030 0.011]	− 0.082	0.064
NWp	− 0.010	[− 0.040 0.015]	− 0.121	0.092
TWp	− 0.012	[− 0.031 0.006]	− 0.078	0.054

Agreement is represented with the bias and limits of agreement for the various tasks

NW03, Normal walking 0.3 m/s; TW03, Target walking 0.3 m/s; NW05, Normal walking 0.5 m/s; TW05, Target walking 0.5 m/s; NW09, Normal walking 0.9 m/s; TW09, Target walking 0.9 m/s; NWp, Normal walking preferred speed; TWp, Target walking preferred speed; CI, confidence interval

## Discussion

### Summary of main findings

This study assessed test–retest reliability and agreement of intramuscular coherence during NW and TW tasks across different walking speeds in healthy individuals. Previous findings of enhanced intramuscular coherence during TW compared to NW were confirmed. Specifically, coherence increased in the high-frequency band for all walking speeds and in the low-frequency band for all, except for the lowest speed with increasing difference at higher walking speeds. These speed-dependent effects were observed in the low-frequency band and, to a lesser extent, in the high-frequency band coherence.

This was the first study to demonstrate inter-trial reproducibility of intramuscular coherence measures and modulation effects induced by TW. This was further confirmed by reliability and agreement measures. The reliability of intramuscular coherence in the Tibialis anterior muscle was moderate to excellent for most NW and TW tasks across all frequency bands. In addition, there were no significant systematic differences in the agreement of intramuscular coherence across sessions and frequencies.

### Task-specific modulation of intramuscular coherence

The TW task induced increased intramuscular coherence compared to NW in the high-frequency band (15–55 Hz) for all walking speeds. This result is in line with previously published studies (Zipser-Mohammadzada et al. 2022; Jensen et al. 2018; Spedden et al. 2019a), and confirms that for preferred or similarly high walking speeds, the TW task represents a challenging walking paradigm that leads to a robust modulation of intramuscular coherence in healthy controls. TW requires attention and precision which in a successful motor performance is dependent on proper propagation of supra-spinal input, as previously shown in visuomotor control studies (Kristeva-Feige et al. 2002; Perez et al. 2006). Consequently, in individuals with iSCI or individuals with other causes of compromised corticospinal interaction, high-frequency coherence is largely absent or reduced (Barthélemy et al. 2010; Hansen et al. 2005; Nielsen et al. 2008; Fisher et al. 2012; Zipser-Mohammadzada et al. 2022). Interestingly, iSCI individuals with residual corticospinal drive (shown by corticospinal activation with TMS) have shown increased intramuscular coherence in the high-frequently band after locomotor training (Norton and Gorassini 2006) and by adapting their gait pattern and controlling of foot placement (Barthélemy et al. 2010). Gait pattern adaptation and controlled foot placement may be reasons iSCI individuals were able to adequately respond to the TW task with increased intramuscular coherence in the high-frequently band to some extent (Zipser-Mohammadzada et al. 2022). It should be emphasized that intramuscular coherence can be used as a proxy for corticospinal input, but does not represent a direct readout of cortical activity. Moreover, other networks, such as spinal circuits (Nielsen et al. 2005) or sensory feedback pathways may play are role in modulating intramuscular and cortico-muscular coherence (Riddle and Baker 2005; Fisher et al. 2002; Kilner et al. 2004).

The increase of intramuscular coherence during TW compared to NW was also observed for the low-frequency band (5–14 Hz) across walking speeds of 0.5 m/s, 0.9 m/s, and preferred speed. These results suggest that the TW task is associated with enhanced overall synchronicity of contributing neural networks during voluntary gait control. This is observed irrespective of speed within a certain range (Zipser-Mohammadzada et al. 2022).

The results presented here imply that the increase of intramuscular coherence during TW cannot be accounted for by a change in walking speed. Furthermore, the lack of significant intramuscular coherence differences between sessions for all tasks in NW and TW suggests that there were no learning effects.

### Modulation of intramuscular coherence during target walking across different speeds

For the slowest walking speed of 0.3 m/s, there was no difference between TW and NW intramuscular coherence in the low-frequency band. TW intramuscular coherence was significantly decreased during TW at 0.3 m/s compared to TW at 0.9 m/s and at preferred walking speed suggesting that the effect of TW on coherence is not completely indifferent of walking speed. One explanation for the results at 0.3 m/s is that this very slow walking speed on a treadmill is similar to a sequence of singular controlled steps being initiated from a standing posture rather than to a continuous locomotor pattern. Hence, slower movements and consecutively longer swing times would allow a subject to accommodate changes in limb- and foot trajectory, thereby altering e.g., feedforward neural control mechanisms and/or sensorimotor integration to resemble each other for TW and NW, different from the condition prevailing at higher treadmill speeds associated with continuous stepping. As a consequence, low-frequency coherence measures during NW and TW at very slow speeds fail to detect a difference of intramuscular coupling.

Furthermore, intramuscular coherence in the low-frequency band is suggested to reflect EMG envelope and gait rhythm features, such as step length, during walking. These effects are mostly attributed to frequencies lower than 8 Hz (Halliday et al. 2003), and the frequency spectrum chosen here, 5–14 Hz, may have captured EMG envelope modulation features during TW, which may explain why stronger speed-specific differences were found in the low-frequency band and less so at higher frequencies. Previously, we did not find a relationship between preferred walking speed and high-frequency coherence effects obtained during TW as compared to NW (Zipser-Mohammadzada et al. 2022). In the latter study, intramuscular high-frequency coherence during TW at the preferred speed was significantly higher than 0.5 m/s, which possibly corresponds to the higher demand for attention during TW at the preferred walking speed.

### Reliability and agreement of intramuscular coherence of the different walking tasks

In the high-frequency band, only the ICC value for TW03 was higher with narrower corresponding 95% CI width compared to NW03. Performing the TW task at a slow walking speed of 0.3 m/s possibly allowed for controlled steps which potentially led to better reliability of intramuscular coherence between sessions 1 and 2. Furthermore, participants may have had time to employ movement strategies that could be applied in the next recording session, i.e., when to lift up, how to move and put down the leg in order to avoid, e.g., double steps between the targets.

Generally, intramuscular coherence in the high-frequency band seems to be more reliable for NW compared to TW. This may be due to a more regular type of walking with consistent stride lengths and -widths which lead to less variety during NW compared to TW.

It should be noted that the reliability and agreement of intramuscular coherence may depend on the type of task and the method employed for data analysis.

For instance, in contrast to the results in this study, Gennaro and de Bruin (2020) found lower reliability of intramuscular Tibialis anterior high-frequency band coherence with a wider 95% CI during walking at a self-selected preferred speed in controls [ICC 0.44 (− 0.23 to 0.87)] compared to the current study [ICC for NW 0.85 (0.62–0.95)]. This is likely related to methodological differences in the challenging walking tasks, i.e., NW and TW were performed on a treadmill while over-ground walking with a figure-8 gait path was performed in the former study. Treadmill walking shows less stride-to-stride variability in spatiotemporal gait parameters compared to straight over-ground walking (Hollman et al. 2016) and entrains a stepping rhythm while figure-8 gait path might additionally have increased variability of steps, thus possibly explaining superior reliability in the present study.

The study from van Asseldonk et al. (2014) also found lower reliability of intramuscular coherence compared to this study. They presented ICC values of 0.76 and 0.47 for walking speeds of 0.7 m/s and 1.4 m/s, respectively, which is much lower than ICC values of 0.97, 0.93, and 0.93 for walking speeds of 0.5 m/s, 0.9 m/s, respectively, and the preferred speed similar to 0.9 m/s in this study. These discrepancies might be attributed to differences in data analysis, for instance, the definition of the swing phase. Furthermore, this present study had a larger sample size and therefore, possibly larger variance between participants’ coherence. If little variation between participants is present, the measurement error will have a more significant negative effect on reliability compared to when there is a greater variation between participants (Hernaez 2015). Hence, a greater variance between participants in the current study could partially explain the high reliability values.

### Limitations and outlook

This study only included young adult, healthy individuals who are free from any age-related decline in motor performance. Therefore, the results from this cohort obtained in this study cannot be generalized to individuals with neurological conditions or older adults. Age-dependent effects were previously demonstrated for reliability of intramuscular coherence in the Tibialis anterior muscle during the swing phase of gait, which was lower in older compared to young adults (Gennaro and de Bruin 2020). However, in a large cohort of 92 healthy adults ranging from 22 to 77 years, no systematic age-related changes were found in the intermuscular coherence amplitude (Jaiser et al. 2016). The scope of this study was the reliability of intramuscular coherence across the whole swing phase. Therefore, the swing phase was not divided into different segments (Halliday et al. 2003; Jensen et al. 2018). Our results demonstrate a clear difference between conditions using the complete swing phase. A more fine-grained comparison of the time course of differences in coherence between conditions could be explored using wavelet coherence (Suwansawang and Halliday 2017). On average, the number of segments or gait cycles analyzed were higher for TW tasks than for NW tasks, meaning 64 additional gait cycles were recorded for TWp/TW09 compared to NWp/NW09. The pooled estimates in Fig. 3 take into account the numbers of segments in each record, so records with low numbers of segments are not given disproportionate weight in the overall estimate (Amjad et al. 1997). We cannot exclude an effect of the number of segments on coherence magnitude. However, differences in intramuscular high-frequency coherence between TW and NW were also found for slow walking speeds TW05/TW03 and NW05/NW03 with comparable number of gait cycles. Furthermore, within the tasks TW and NW, the number of segments or analyzed gait cycles differed from slow to fast speeds but no systematic increase of coherence was obtained for the high-frequency range.

Furthermore, participants performed each task only once per session. Therefore, reliability and agreement analysis could only be performed on a group level. However, it would be favorable to further investigate variability and reproducibility of intramuscular coherence effects such as induced by TW or training within the individual subjects rather than on a group level for many applications in clinical and research settings. In order to substantiate the results on reliability and agreement, a larger sample size would be needed in future studies (Koo and Li 2016) where inclusion of older adults would provide more comparative data for typical clinical populations of interest in this field.

## Conclusions

To our best knowledge, this is the first study investigating the reliability and agreement of intramuscular EMG coherence effects during a challenging visually guided compared to a normal walking task across a range of different walking speeds. The results show that intramuscular coherence increase in the low-frequency (5–14 Hz) and high-frequency band (15–55 Hz) during the swing phase is a reliable and reproducible marker of visually guided motor control and is thus suggested to allow to asses features of volitional control of gait in young adults. Moreover, the consistent increase of intramuscular coherence during TW compared to NW across the frequency bands and walking speeds suggests that TW is associated with an increased requirement for common central drive to the leg muscles. This represents additional evidence that features of intramuscular coherence are dependent on supra-spinal input. It suggests that the assessment of intramuscular coherence in different walking tasks may be a promising and insightful assessment on the functional integrity of supra-spinal control in neurological conditions. The longitudinal investigation of intramuscular coherence may thus help to quantify treatment and rehabilitation effects in neurological conditions.

## Supplementary Information

Below is the link to the electronic supplementary material.

Supplementary file 1 (DOCX 199 KB)

## Data Availability

All data generated or analyzed during this study are included in this published article and its supplementary information files.
