# Supplementary material for: Intramuscular coherence enables robust assessment of modulated supra-spinal input in human gait: an inter-dependence study of visual task and walking speed
Source: Exp Brain Res. 2023 May 18;241(6):1675–89. doi: 10.1007/s00221-023-06635-4 (PMC10224862; doi:10.1007/s00221-023-06635-4)
Supplement: Supplementary file 1 — (DOCX 199 KB) [file 221_2023_6635_MOESM1_ESM.docx]

**Intramuscular coherence enables robust assessment of modulated supra-spinal input in human gait. An inter-dependence study of visual task and walking speed**

Experimental Brain Research

Freschta Zipser-Mohammadzada*^#1^, Marjelle Fredie Scheffers*^1,2^, Bernard A. Conway^3^, David M. Halliday^4,5^, Carl Moritz Zipser^1^, Armin Curt^1^, Martin Schubert^1^

**# Correspondence:**

Freschta Zipser-Mohammadzada

Spinal Cord Injury Center, Department of Neurophysiology, Balgrist University Hospital, Zurich, Switzerland, fzipser.moh@gmail.com

In this study, the pooled coherence was calculated for each task in each session, sessions 1 and 2 (i.e., test and retest) to obtain differences between tasks and between sessions at a population level. The $\chi^{2}$ extended difference of coherence test (Amjad et al., 1997) was used to explore differences at each frequency between tasks at both sessions and between sessions 1 and 2 within each task. As depicted in the figure below at the preferred walking speed and at 0.9 m/s, significant differences between the tasks Normal and Target walking can be found across a broad frequency spectrum, from 5-60 Hz (p<0.001). However, at 0.5 m/s, significant differences between the tasks are found between 5-12 Hz, 13-18 Hz, and 20-40 Hz (p<0.05), whereas the differences are mostly abolished at 0.3 m/s. The results indicate that the significant increase of Target walking coherence from Normal walking coherence is consistent across sessions and reduced at slow walking speeds.

Between sessions 1 and 2 at preferred and 0.9 m/s walking speed, significant peaks for Target walking are seen at isolated frequencies, rather than consistently over the frequency range 1-50 Hz as seen when comparing Target and Normal walking. Significant differences at single frequencies can occur due to chance (Amjad et al., 1997). Similar effects are observed at the slow walking speeds 0.5 m/s and 0.3 m/s. Thus, intramuscular coherence does not differ significantly over a large frequency range, suggesting it is therefore reproducible.


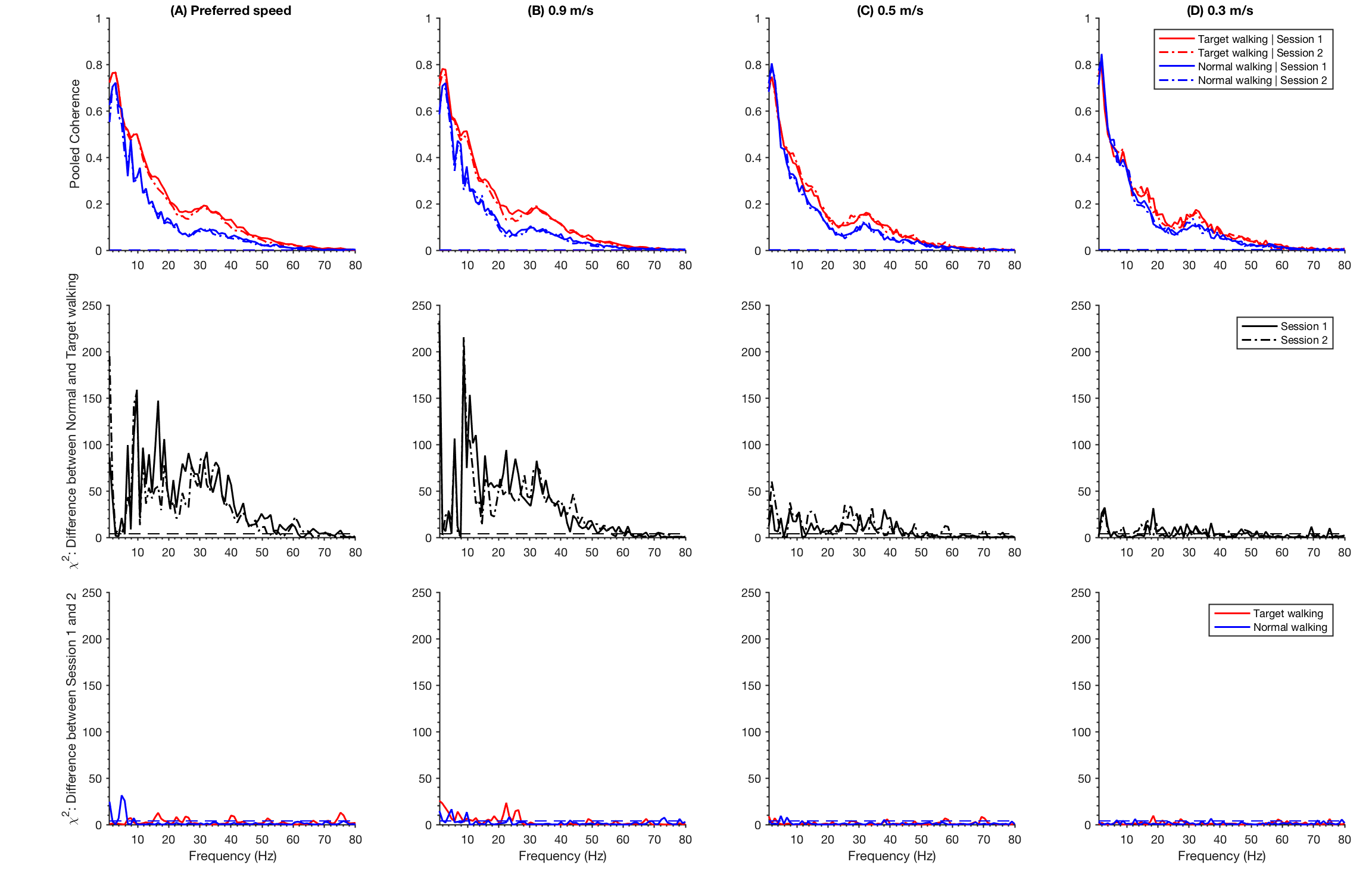


AMJAD, A. M., HALLIDAY, D. M., ROSENBERG, J. R. & CONWAY, B. A. 1997. An extended difference of coherence test for comparing and combining several independent coherence estimates: theory and application to the study of motor units and physiological tremor. *J Neurosci Methods,* 73**,** 69-79.
